# PET-CT staging of the neck in cancers of the oropharynx: patterns of regional and retropharyngeal nodal metastasis

**DOI:** 10.1186/1477-7819-8-70

**Published:** 2010-08-16

**Authors:** Marcie Tauzin, Amy Rabalais, Joseph L Hagan, Charles G Wood, Robert L Ferris, Rohan R Walvekar

**Affiliations:** 1 Department of Otolaryngology Head Neck Surgery, LSU Health Sciences Center, New Orleans, LA, USA; 2 Biostatistics program, School of Public Health, LSU Health Sciences Center, New Orleans, LA, USA; 3 Mary Bird Perkins Cancer Center, Baton Rouge, LA, USA; 4 Department of Otolaryngology Head Neck Surgery, University of Pittsburgh, Pittsburgh, PA, USA

## Abstract

**Objective:**

To study the retropharyngeal lymph node status (RPLN) by pretreatment PET-CT imaging in patients with squamous cell carcinomas of the oropharynx (OPSCC). Study Design: Retrospective.

**Methods:**

101 patients with a biopsy proven OPSCC were identified. 53 patients meeting inclusion criteria were further analyzed.

**Results:**

The frequency of RPLN was 20.8% (11/53). Advanced T stage cancer (OR = 5.6250, 95% CI: 1.06 - 29.80, p = 0.0410) and advanced clinical N stage cancer (i.e. N2+) had higher odds (OR = 3.9773, 95% CI: 0.9628 - 16.4291) of being RPLN positive as compared to N0-1 patients.

**Conclusions:**

Pre-treatment PET-CT can be used as a staging tool to aid in treatment planning of OPSCC, as rates of RPLN and nodal metastasis are consistent with those reported in the literature. Advanced T and N stage are associated with a greater odds ratio of being RPLN positive by PET-CT imaging.

## Introduction

Over the last two decades there has been a gradual shift in the presentation of OPSCC, with an increased incidence in a younger patient population[1,2]. Radiotherapy or chemoradiotherapy has been advocated as the treatment of choice for oropharyngeal squamous cell carcinoma (OPSCC) to avoid morbidity of traditional surgical resection [3,4]. However, both radiotherapy and chemoradiotherapy also have known severe local adverse effects and systemic toxicities. Additionally, treatment with radiotherapy eliminates its future use for management of second head and neck primary cancers (up to 25%) [2,5]. Traditional surgical options for OPSCC are not considered the treatment of choice for management of these tumors due to equivalent survival outcomes with chemoradiation and also due to associated morbidity with large open resections. However studies have shown that cancers of the oropharynx, if limited in nature (e.g. T1-2, N0-1), can be offered minimally invasive surgical therapy which has lower morbidity and equivalent margin control, as compared to traditional surgical options, while also preserving non- surgical treatment options for the future management of second primary cancers and/or recurrent tumors [1,6].

Although primary tumor control is achievable in early tumors with minimally invasive surgery, such as transoral or robot-assisted procedures, the management of the neck is still an important consideration in the treatment of OPSCC. Surgical management of the neck in patients with OPSCC does not usually involve a dissection of the RPLNs. However, when RPLNs are treated surgically, the RPLN dissection is in conjunction with primary resection and standard neck dissection in patients with advanced carcinoma of the oropharynx and hypopharynx. RPLN dissection involves resection of this nodal basin up to the skull base along with the primary site in an en bloc fashion, using a mandibular-splitting procedure in most cases [7-9]. This approach divides the small nerves of the pharyngeal plexus in the process of separating the pharyngeal wall from the structures of the carotid sheath and can be associated with increased severity of dysphagia [10]. Neck dissections do not routinely address RPLNs; creating a potential for recurrence in the retropharynx and the need to address this nodal basin with radiotherapy. This has been one of the criticisms of primary surgical treatment for OPSCC.

Thus, in order to individualize treatment strategies for patients, pre-treatment information regarding the status of RPLNs would be important. The status of the RPLN involvement with cancer prior to treatment planning would be helpful in selecting patients who may benefit from surgical therapy or staging, i.e. patients with early-intermediate (T1-2;N0-1) stage OPSCC without RPLN involvement[2]. The decision to treat with surgery only, post-operative radiation, or post-operative chemoradiation therapy could then be individualized after evaluating the surgical specimen pathologically. We have previously proposed this treatment algorithm in a prior study [2]. This study demonstrated that surgical staging in limited OPSCC can identify patients in whom intensification of treatment with chemotherapy can be most appropriately applied, and conversely enables de-intensification of therapy in pathology confirmed stage I-II disease.

The introduction of PET-CT imaging for assessment of cancers of the head and neck revolutionized cancer staging and is now routinely performed prior to planning therapy. However, few studies have evaluated regional nodal distribution and RPLN assessment by PET-CT imaging for OPSCC. Our aim was to study the distribution of regional lymphadenopathy and RPLN status via pretreatment PET-CT imaging.

## Materials and methods

Institutional Review Board approval was obtained before initiating this retrospective chart review. A hundred and one patients treated at Mary Bird Perkins Cancer Center (MBPCC) between September 2002 and March 2008, with biopsy proven squamous cell carcinoma of the oropharynx were identified by searching the MBPCC patient data base with appropriate ICD-9 codes. All patients received primary non-surgical therapy. The inclusion criteria are listed in Appendix 1. Fifty-three patients meeting inclusion criteria were further analyzed for this study after excluding 48 from the analysis for reasons outlined in Table 1. Fourteen patients with early and intermediate stage OPSCC who underwent primary surgical therapy were referred to MBPCC for post-operative radiation or chemoradiation. These patients had planning PET-CT scans at MBPCC after initial surgical therapy; therefore, due to lack of pre-treatment staging PET-CT they were excluded from the study.

**Table 1:** Exclusion Criteria

Insufficient data in chart	8
Primary surgical therapy	14
Lost to follow up	5
Did not receive treatment at MBPCC	14
Metastatic disease at presentation	5
Palliative treatment only	1
No pre-treatment PET-CT	2
Total number of excluded patients	48

Demographic data, clinical data that included findings at physical examination, staging information, and pretreatment staging positron emission tomography-computerized tomography data that included features of the primary tumor and regional metastasis was recorded in all cases. In addition to a review of the initial radiology report, all pretreatment staging PET-CT scans were re-evaluated with emphasis on evaluating retropharyngeal lymphadenopathy. Mean standard uptake values (SUV) and size (in cm) of all FDG avid lesions including primary tumor site and all metastatic lymph nodes were recorded. PET-CT scans were reviewed for regional metastatic lymphadenopathy, which was quantified as ipsilateral or contralateral levels I-V lymphadenopathy. The cutoff mean SUV was 3.0, with mean SUVs of less than 3.0 defined as negative and mean SUVs of greater than 3.0 defined as positive [11]. However, because SUVs are semi-quantitative, it is not possible to determine the specific value for reference [11]. All nodal metastasis was deemed positive if the measured cut-off value was greater than or equal to 1.0 cm or any suspicious features such as central necrosis were present [12,13]. Lesions that met either the size and/or mean SUV criteria defined above were considered "positive".

All scans were PET-CT fusion studies and were obtained on a single scanner at MBPCC in the majority of cases (47/53). PET-CT scans were obtained in standard protocol as previously described in a prior study from our institution [14].

Follow up data was obtained on all patients in the study cohort at one month post treatment and at last follow-up. Data recorded at last follow-up included information on loco-regional recurrence and distant metastasis and treatment of the same. Statistical analyses were performed using the Wilcoxon-Mann-Whitney U test, exact Pearson Chi-Square test, and odds ratios.

## Results

### Demographic data

The mean age of the study population was 57.2 years (range, 41-88 years) with a male to female ratio of 46:7. The most common site for cancer within the oropharynx was the tonsil (62.4%; 33/53) followed by base of tongue (26.4%; 14/53). The incidence of tumor, nodal, and overall stages for the cohort is listed in Table 2.

**Table 2:** Tumor and nodal status and clinical stage for the study cohort

Tumor Stage			Nodal Stage		
T1	1/53	1.9%	N0	21/53	39.6%
T2	21/53	39.6%	N1	8/53	15.1%
T3	24/53	45.3%	N2a	12/53	22.6%
T4	7/53	13.2%	N2b	4/53	7.6%
Early Stage (I or II)	7/53	13.2%	N2c	7/53	13.2%
Late Stage (III or IV)	46/53	86.8%	N3	1/53	1.9%

### Treatment details

All patients were treated with intensity-modulated radiation therapy (IMRT) at MBPCC with an average dose to the primary tumor, retropharynx, and gross disease in the neck of 69 Gy (range, 64-72Gy). Forty-seven patients (88.7%) received concurrent chemotherapy, 3 patients (5.7%) additionally received neoadjuvant chemotherapy, and 1 patient (1.9%) also received post- radiation chemotherapy. Five patients (9.4%) did not receive any chemotherapy. Patients were then followed on average for 26 months (range, 1.6 to 63 months).

### PET-CT results

Patients had pre-treatment PET-CT scan on average 2.5 weeks (range, 0.5--15.8 weeks) prior to treatment. The primary tumor size was on average 4.4cm(range, 1.4-12cm) and average SUV of 12.1(range, 2.1-31). The distribution of ipsilateral lymphadenopathy and contralateral lymphadenopathy are illustrated in Table 3.

**Table 3:** Nodal distribution by PET-CT

Distribution	Ipsilateral Lymphadenopathy		Contralateral Lymphadenopathy	
**Level**	**Frequency**	**Percent**	**Frequency**	**Percent**

Level I	2	3.8%	1	1.9%
Level II	40	75.5%	16	30.2%
Level III	23	43.4%	2	3.8%
Level IV	5	9.4%	0	0%
Level V	2	3.77%	0	0%

PET-CT upstaged approximately 43.4% when compared to clinical stage, down staged 5.7%, and did not change the stage in 50.9%. Examination of the impact PET-CT scan has on clinical stage indicated that there was not a significant difference (p = 0.2961) in the T stage of subjects with upstage compared to those who were not upstaged by PET-CT. However, there was a trend toward overall nodal upstaging by PET-CT scan when compared to clinical stage.

### Distribution of regional metastasis by PET-CT

The regional metastatic nodal distribution of late stage (III and IV) tumors was 10.8% (5/46) N0, 15.2% (7/46) N1, 10.8% (5/46) N2a, 32.6% (15/46) N2b, 26.1% (12/46) N2c, 4.8% (2/46) N3. Table 3 illustrates that the greatest concentration of regional metastasis was in level II distribution regardless of laterality.

### Retropharyngeal Lymphadenopathy

The frequency of retropharyngeal lymphadenopathy in this cohort was 11 of 53 patients (20.8%). Of the 11 patients with retropharyngeal lymphadenopathy, 82% (9/11) had T3 disease while 18% (2/11) had T2 disease. Of the seven patients with T4 disease, 57% (4/7) had direct invasion of the retropharynx with the primary tumor, making a distinction between the primary tumor and RPLN imprecise. Thus, these patients were not included in this analysis. RPLN positivity was significantly associated with T stage, (*X*^2 ^= 24.88, df = 6, p = 0.0003). Subjects with advanced T stage cancer (i.e. T3 or T4 tumors) have significantly higher odds (OR = 5.6250, 95% Confidence Interval: 1.06 - 29.80, p = 0.0410) of being RPLN positive by PET-CT.

The regional nodal status of the 11 patients with RPLN was N0 (1/11), N1 (0/11), N2a (2/11), N2b (4/11), N2c (4/11), and N3 (0/11). Similarly there was a significant association (*X*^2 ^= 25.9535,df =10 p = 0.0045) between RPLN positivity and N Stage. Subjects with more advanced clinical N stage cancer (i.e., N2a, N2b, N2c and N3) have higher odds (OR = 3.9773, 95% Confidence Interval: 0.9628 - 16.4291) of being RPLN positive by PET as compared to those with early clinical N stage; however, the association misses the 0.05 cutoff for statistical significance (p = 0.0765).

The presence of retropharyngeal lymphadenopathy was stratified by primary tumor site. The most common site was tonsil (82%, 9/11) with the remaining sites posterior pharyngeal wall and the base of tongue each 9% (1/11). Of all tonsil primary tumors, 27.3% (9/33) had retropharyngeal lymph node involvement. Similarly, 28.5% (4/14) base of tongue primary tumors and 20% (1/5) posterior pharyngeal wall tumors had retropharyngeal lymphadenopathy.

### Patterns of recurrence

The overall recurrence rate was 35.8% (19/53) with 7.5% (4/53) local recurrence, 13.2% (7/53) regional recurrence, and 15.1% (8/53) distant metastatic disease. One patient (1.9%, 1/53) was diagnosed with a second primary tumor.

## Discussion

The incidence of RPLN metastasis identified by PET-CT in our OPSCC cohort was 20.8%. This is well within the reported range of 16-50% published in previous studies [7,8,10,15]. We found that advanced T stage cancer (T3 - T4) have significantly higher odds of being RPLN positive by PET-CT scan. Shimizu conducted a study where RPLNs were electively dissected in cases where the primary tumor originated from or invaded the posterior or lateral wall of the oropharynx [9]. Histology confirmed RPLN metastatic distribution was similar to our findings of PET-CT distribution where the majority (60%) were T3 and T4 tumors[9].

The epicenter of RPLN metastasis in OPSCC has been most commonly from tonsillar primary tumors, followed by posterior pharyngeal wall tumors, then base of tongue tumors [9]. Similarly, our series demonstrates tonsillar primary tumor sites to be the most common source of RPLN metastasis; however, we have found that posterior pharyngeal wall and base of tongue tumors had equal predilection for metastasis to RPLNs. These findings can be attributed to the small sample size of our study.

The ability of PET-CT to accurately assess and be in accordance with prior studies of metastatic lymph node distribution is imperative for treatment planning of the N0 neck irrespective of the modality chosen. As previously reported, lymph node metastasis of OPSCC is most commonly seen at levels II and III [9,16,17]. PET-CT findings in this study are in agreement with this data confirming that PET-CT could accurately detect nodal disease in the staging of head and neck cancer patients. Furthermore, several studies have already shown that adding PET- FDG or PET/CT-FDG to standard work-up led to a higher staging accuracy with higher specificity [18-21].

A recent study examining this issue was conducted by Lonneaux et al in a prospective, multicenter study showing that PET-FDG was significantly more accurate than conventional staging (McNemar test, P < .0001) and improved staging accuracy in 20% of patients with head and neck squamous cell carcinoma [21]. Furthermore, they showed PET-FDG imaging modified the management of 13.7% of patients. Our findings indicate that PET-CT changed the stage in a large number of patients: upstaging approximately 43.4% and down staging 5.7% when compared to clinical stage. While our retrospective study did not examine the impact this had on treatment strategies (i.e. change in radiation fields or doses), it raises the question as to how this potentially affects patient outcomes. Lonneux et al findings significantly contribute to growing body of knowledge that PET-CT is an important tool in pre-treatment work-up and should be implemented as routine imaging of head and neck squamous cell carcinoma [21]. Additionally, our study highlights the importance and usefulness of pre- treatment PET-CT in detecting RPLN status and its role in guiding definitive treatment in OPSCC.

Our findings also showed a significant association between N stage and positive RPLN status. Patients with N2 or greater nodal disease on clinical presentation had higher odds of having positive RPLN status by imaging criteria as compared to those patients who presented with N0-1 disease.

The small sample size is a limitation of our study. However, our observations are consistent with radiologic and histologic studies reported in the literature.

## Conclusion

PET-CT results for OPSCC can be used as a staging tool to aid in treatment planning, as rates of RPLN and nodal metastasis are consistent with those reported in the literature. Advanced T and N stage are associated with a greater odds ratio of being RPLN positive by PET-CT imaging.

## Competing interests

The authors declare that they have no competing interests.

## Authors' contributions

MT conducted the chart review, data collection, contributed to study design and study co-ordination. AG participated helped in chart review, data collection and organization and also helped to edit the manuscript. JH performed statistical analysis. CW participated in study design and editorial contributions to the manuscript. RF contributed to the study design and also editorial contributions. RW conceived of the study, and participated in its design, write up, data analysis, editorial changes, and coordination as the corresponding author. All authors read and approved the final manuscript.

## Appendix 1: Inclusion Criteria

• Biopsy proven diagnosis of SCC* of the oropharynx

• Primary non-surgical therapy

• Sufficient medical record documentation

• Pre-treatment PET-CT scan

• Received all therapy at MBPCC**

• No evidence of metastatic disease at presentation

*SCC: Squamous cell carcinoma; **MBPCC: Mary Bird Perkins Cancer Center
